# Supplementary material for: PDE10A Inhibition Reduces NLRP3 Activation and Pyroptosis in Sepsis and Nerve Injury
Source: Int J Mol Sci. 2025 May 8;26(10):4498. doi: 10.3390/ijms26104498 (PMC12111586; doi:10.3390/ijms26104498)
Supplement: Supplementary file 1 [file ijms-26-04498-s001.zip › ijms-3576172-supplementary.pdf]

Supplemental data

PDE10A Inhibition Reduces NLRP3 Activation and Pyroptosis in Sepsis and Nerve Injury

Bradford C. Berk [1,2,*], Camila Lage Chávez [1] and Chia George Hsu [1,3,*]

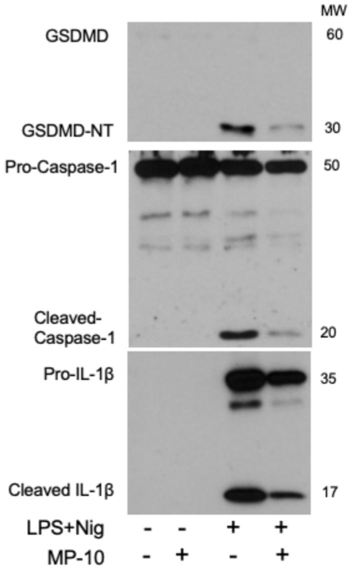

Supplemental Figure S1. PDE10A inhibition reduces NLRP3 inflammasome activation in bone marrow-derived macrophages (BMDMs). BMDMs were stimulated with LPS (100 ng/mL) for 3 hours, with or without MP-10 (5 μM), followed by treatment with nigericin (6 μM). Pro- and cleaved caspase-1, IL-1β, and GSDMD levels were measured by western blotting.
